# Pharmacokinetics of micafungin in patients with severe burn injuries

**DOI:** 10.1186/cc9661

**Published:** 2011-03-11

**Authors:** J Sasaki, S Kishino, S Hori, N Aikawa

**Affiliations:** 1 Keio University School of Medicine, Tokyo, Japan; 2 Meiji Pharmaceutical University, Tokyo, Japan

## Introduction

Micafungin (MCFG), an echinocandin antifungal agent, exhibits more potent antifungal activity against a broad spectrum of clinically important Candida and Aspergillus species [1]. However, there are few pharmacokinetic data of antifungal agents for burned patients, and determination of the dosage for these populations requiring initially a large quantity of fluid therapy can trouble burn surgeons and intensivists. The purpose of this study is to obtain the pharmacokinetic data for MCFG in severe burned patients.

## Methods

In six patients with severe burn injuries within 14 days after injuries (19 to 82 years old, 36 to 85% TBSA), we measured the plasma concentration of MCFG by high-performance liquid chromatography [2] after drip infusion of MCFG, at 200 to 300 mg/day over a 1-hour period. Blood samples were collected at the end of the initial administration of MCFG (peak value after initial administration; A point), immediately before the second dosing (trough value after initial administration; B), at the end of the fourth dosing (steady-state peak value; C), and immediately before the fifth dosing (steady-state trough value; D). The control value of plasma concentration of MCFG assumed the pharmacokinetics value obtained from healthy volunteers.

## Results

**Figure 1:**
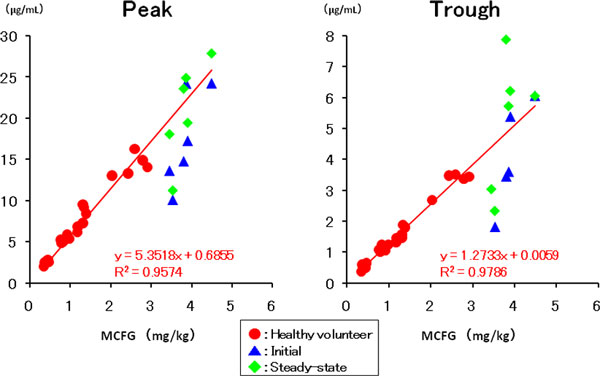
**Correlation between the plasma concentration of MCFG and the dose of MCFG (mg/kg)**.

The plasma concentration of MCFG at the A point were 10.1 to 24.2 μg/ml, 1.8 to 6.1 μg/ml at B, 11.3 to 27.9 μg/ml at C, and 2.3 to 7.9 μg/ml at D. In both peak and trough values there was a good correlation between the plasma concentration of MCFG and the dose of MCFG per kilogram body weight the same as cases of healthy volunteers (Figure 1).

## Conclusions

These results suggest that MCFG can be administered safely to burned patients without adjusting the dose.
